# Novel sustainable biodiesel production from low-grade oleic acid via esterification catalyzed by characterized crystalline ZrO_2_/Al_2_O_3_

**DOI:** 10.1186/s13065-024-01360-7

**Published:** 2025-01-04

**Authors:** Amal Alkahlawy, Amany Gaffer

**Affiliations:** 1 Refinery department, Egyptian Petroleum Research Institute (EPRI), Nasr City, Cairo, 11727 Egypt; 2 Petroleum Application Department, Egyptian Petroleum Research Institute (EPRI), Cairo, 11727 Egypt

**Keywords:** Heterogeneous catalyst, Biodiesel production, Catalytic esterification

## Abstract

The depletion of fossil fuels and growing environmental concerns necessitate the exploration of renewable energy sources. Biodiesel, a promising alternative fuel derived from sustainable feedstock, has attracted considerable attention. This study investigates the catalytic esterification of oleic acid, a readily available fatty acid, with ethanol for biodiesel production using a novel heterogeneous catalyst, ZrO_2_/Al_2_O_3_. Crystalline ZrO_2_/Al2O_3_ was successfully synthesized and characterized using X-ray diffraction (XRD), Brunauer–Emmett–Teller (BET) surface area analysis, and Fourier-transform infrared spectroscopy (FTIR), X-ray photoelectron spectroscopy (XPS) and temperature programmed desorption NH_3_-TPD to understand its structural and textural properties. The characterized ZrO_2_/Al_2_O_3_ was then employed to catalyze the esterification reaction. The influence of reaction parameters, including temperature, alcohol-to-oleic acid molar ratio, and catalyst loading, was systematically evaluated. Under optimal conditions (70 °C, 10:1 alcohol-to-oleic acid molar ratio, and 4 wt% catalyst loading), a remarkable 90.5% conversion of oleic acid to biodiesel was achieved. Furthermore, the catalyst exhibited reusability, demonstrating its potential for sustainable biodiesel production from low-grade oleic acid feedstock.

## Introduction

The escalating energy demand coupled with dwindling fossil fuel reserves necessitates exploring renewable energy sources. Biodiesel, derived from biological sources like vegetable oils and animal fats, is a promising alternative to conventional fossil fuels because of its biodegradability, non-toxicity, potential to reduce greenhouse gas emissions, and potential for domestic production [1–3]. A key pathway for biodiesel synthesis involves the catalytic esterification of free fatty acids (FFAs), such as the abundant oleic acid, with short-chain alcohols like ethanol. This reaction converts triglycerides in the feedstock into fatty acid ethyl esters (FAEEs), the primary components of biodiesel [4–7].

However, conventional methods often rely on homogeneous catalysts, which pose challenges in separating the product and recovering the catalyst for reuse [8].

To overcome these limitations, heterogeneous catalysts offer a promising alternative [9–11]. Existing in a separate phase from the reactants, they allow for easier separation through filtration or centrifugation and potential reusability, leading to reduced environmental impact and production costs.

Metal oxides are particularly promising due to their abundance of acidic and basic sites, allowing them to be reused without significant activity loss [12–17]. Common types of heterogeneous solid acidic catalysts include zeolites, Nafion-NR50, tungstate zirconia (WO_3_/ZrO_2_), lanthanum oxide (La_2_O_3_), and sulfated zirconia [18].

Among the promising candidates for heterogeneous biodiesel production are ZrO_2_/Al_2_O_3_ composites [19, 20]. These composites combine the beneficial properties of both components, ZrO_2_ contributes Lewis acid sites that activate reactants, while Al_2_O_3_ provides structural stability, a high surface area with numerous active sites, and thermal stability. This combination facilitates efficient reactant adsorption and promotes the esterification process [19, 21–23].

Previous studies, such as those by Xie et al., have explored the use of solid catalysts in biodiesel production, employing magnetic solid catalysts (Fe_3_O_4_/MCM-41) [24] and hierarchical porous catalysts (SAPO-34) [25]. These studies achieved high oil conversion yields. Inspired by these findings, we chose to investigate the ZrO_2_/Al_2_O_3_ composite for its potential to optimize reaction conditions, evaluate conversion efficiency and reusability, and contribute to a more sustainable and cost-effective biodiesel production process using low-grade feedstock.

The primary goal of this research is to develop a highly effective solid catalyst for biodiesel production. Specifically, we aim to investigate the potential of ZrO_2_/Al_2_O_3_ composites as heterogeneous catalysts for converting low-grade oleic acid into biodiesel through esterification with ethanol.

This study introduces several key innovative aspects. First, the catalyst composition leverages the combined effects of ZrO_2_ and Al_2_O_3_. ZrO_2_ provides essential Lewis acid sites for effective reactant activation, while Al_2_O_3_ offers structural stability and a high surface area with numerous active sites, resulting in improved catalytic performance.

Secondly, unlike many studies that rely on high-purity feedstock, we specifically target low-grade oleic acid, which is more abundant and cost-effective. This approach reduces raw material costs and aligns with sustainable and environmentally friendly principles of biodiesel production. Through extensive experimentation, we have optimized the reaction conditions, achieving a remarkable conversion of oleic acid to biodiesel under relatively mild conditions.

Additionally, our research highlights the reusability of the ZrO_2_/Al_2_O_3_ catalyst, maintaining its activity over multiple cycles. This reusability is crucial for industrial applications as it significantly reduces overall production costs and environmental impact. A comprehensive characterization of the ZrO_2_/Al_2_O_3_ catalyst has been conducted, providing detailed insights into its structural and chemical properties. This is essential for optimizing its performance and ensuring its effectiveness in biodiesel production.

## Experiment

### Materials

Absolute ethanol (CH_3_CH_2_OH), phenolphthalein (95%), Ammonia solution, Oleic acid), toluene (99.5%), Hydrated aluminum nitrate (Al (NO_3_)_3_.9H_2_O), Hydrated zirconium oxychloride (ZrOCl_2_.8H_2_O), Potassium Hydroxide (KOH) were obtained from Sigma-Aldrich. All the chemicals used were of analytical grade, of 99.9% purity.

### Catalyst preparation

The sol-gel technique offers a versatile approach for synthesizing ZrO_2_/Al_2_O_3_ catalysts. The process begins with hydrated aluminum nitrate (Al (NO_3_)_3_·9H_2_O) and hydrated zirconium oxychloride (ZrOCl_2_·8H_2_O) as precursors. With continuous stirring, a 1:1 NH_3_ solution was added dropwise to the precursor solution maintained at room temperature (27 °C). The obtained hydrogel undergoes an aging process for 24 h at ambient temperature. The aged gel is repeatedly washed with boiling distilled water and filtered to remove residual chloride (Cl⁻) and nitrate (NO₃⁻) ions introduced by the precursors. After washing, the gel is dried using appropriate methods, such as low-temperature oven drying, to remove the solvent (water) trapped within the gel network. The dried gel is then calcined in a muffle furnace at a high temperature of 900 °C for 4 h in static air. This calcination process serves two purposes, it removes any remaining organic residues left over from the precursors, and it converts the amorphous metal precursors into crystalline ZrO_2_ and Al_2_O_3_ phases [26]. 

#### Characterization of ZrO_2_/Al_2_O_3_ catalyst

The structural and morphological properties of the synthesized ZrO_2_/Al_2_O_3_ catalyst were investigated using various techniques:

- X-ray Diffraction (XRD) utilizes a Shimadzu diffractometer with Cu Kα radiation (λ = 1.5406 Å) at a set voltage (e.g., 40 kV) and current (e.g., 40 mA) to analyze the crystal structure of the catalyst. The XRD pattern provides information about the phases present in the material and their relative abundance.
- Fourier-Transform Infrared Spectroscopy (FTIR) using a spectrometer like Thermo Fisher Scientific Nicolet iS™ 10, FTIR spectroscopy helps identify the functional groups present on the ZrO_2_/Al_2_O_3_ surface. The analysis reveals the presence and types of chemical bonds within the catalyst structure.
- Brunauer-Emmett-Teller (BET) Surface Area Analysis using equipment like USA-NOVA 3200, determines the specific surface area and pore volume of the ZrO_2_/Al_2_O_3_ catalyst. These properties influence the number of reactants that can interact with the catalyst and contribute to its overall catalytic efficiency.
- X-ray photoelectron spectroscopy (XPS) using a Thermo Fisher ESCALAB250Xi instrument was employed to determine the chemical state of the elements in ZrO_2_/Al_2_O_3_.
- Temperature programmed desorption NH_3_-TPD- TPDRO/1100 for determining the nature and the total acid of ZrO_2_/Al_2_O_3_.

#### Catalytic esterification test

The catalytic activity of the synthesized ZrO_2_/Al_2_O_3_ catalyst was evaluated for the esterification of oleic acid with ethanol. The reaction was conducted in a 100 mL glass reactor equipped with a magnetic stir bar coated with Teflon and connected to a condenser. Before the reaction, the ZrO_2_/Al_2_O_3_ catalyst was activated by placing it in an air-drying oven at 130 °C. This thermal treatment removes any adsorbed material on the catalyst surface, ensuring optimal activity during the esterification process. Around 50 mL of oleic acid was added to the reactor. The oil bath temperature was then raised to a predetermined value for the reaction. After reaching the desired temperature, a specific amount of the pre-treated ZrO_2_/Al_2_O_3_ catalyst was introduced to the reaction mixture. The catalyst amount was expressed as a percentage of the total oleic acid mass (e.g., 1%, 3%, or 5%). Ethanol was subsequently added to the reaction mixture in a 6:1 molar ratio relative to oleic acid. This ensures sufficient ethanol availability for efficient esterification. The reaction was investigated under various temperature settings (60 °C, 70 °C, and 80 °C) and different catalyst loadings (1%, 3%, and 5%). To monitor the reaction progress, 5 mL samples were periodically withdrawn from the reaction mixture every 15 min. These samples were then analyzed to determine the conversion of oleic acid to the desired ester product. The conversion of oleic acid to ester was determined using an acid-base titration method. The withdrawn samples were first centrifuged at 3000 rpm for 10 min to separate the catalyst residue and water produced during the reaction. The remaining liquid phase was then titrated with a potassium hydroxide (KOH) solution to determine the remaining acid content.

The acid value (AV) was calculated using the following equation:

Acid Value (mg KOH/g) = V × 0.1 × 56.1 M.

Where:

V = Volume of consumed KOH solution (mL).

M = Mass of oleic acid in the sample (g).

1. 0.1 = Concentration of KOH solution (M).
2. 56.1 = Molar mass of KOH (g/mol).

The percentage conversion of oleic acid to ester was then calculated using the following equation:

Conversion (%) = (AV t₀ - Avt) / Avt₀ × 100.

Where:

Av t₀ = Initial acid value of the oleic acid.

Av t = Acid value of the reaction mixture after time t.

## Results and discussion

### Catalyst characterization (ZrO_2_/Al_2_O_3_)

**Fig. 1:**
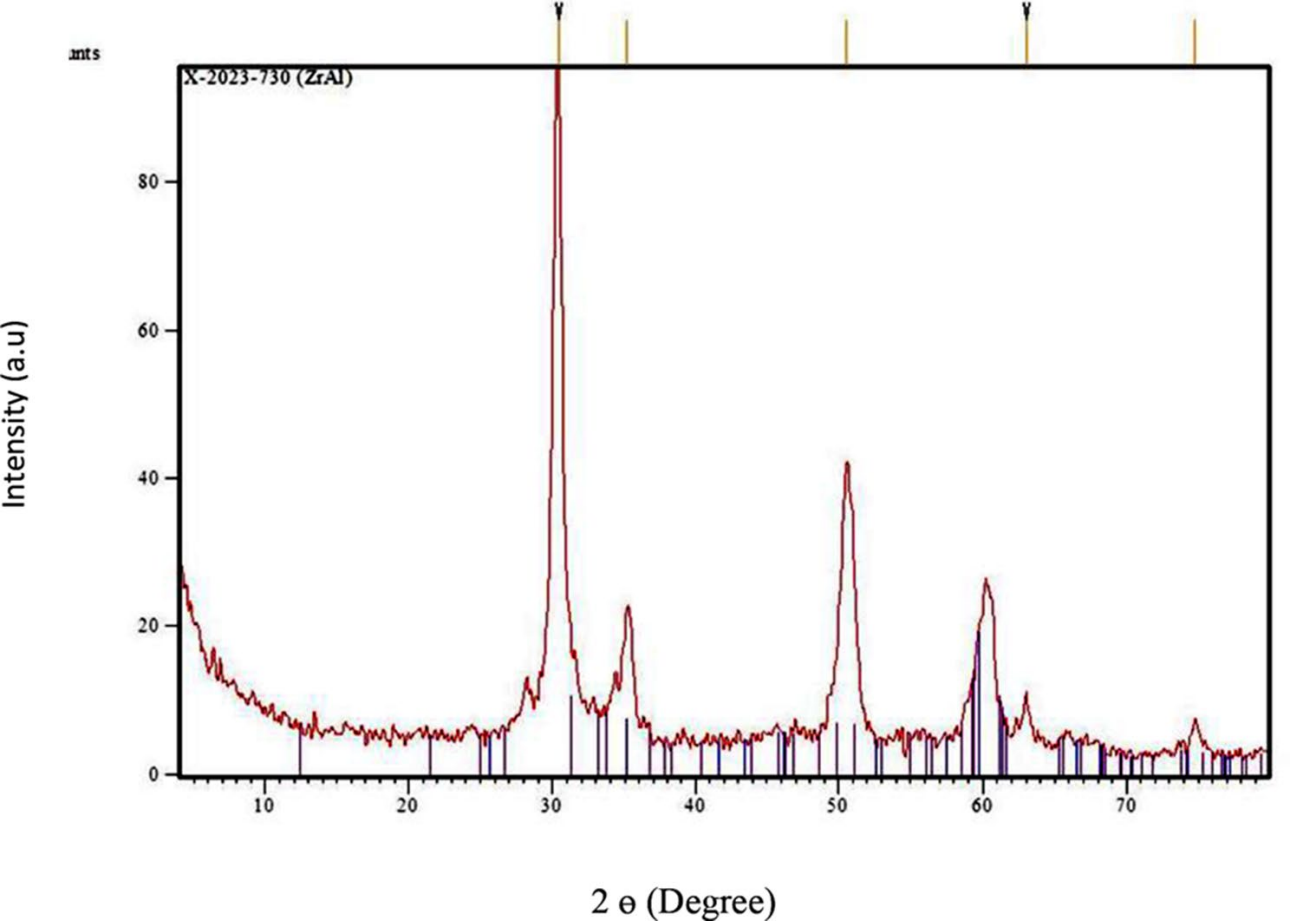
The XRD pattern of ZrO_2_/Al_2_O_3_

The XRD pattern of ZrO_2_/Al_2_O_3_ is shown in Fig. 1 The XRD pattern of the ZrO_2_/Al_2_O_3_ catalyst exhibits characteristic peaks at 2θ = 30.2°, 50.2°, and 60.1° corresponding to the tetragonal phase of ZrO_2_, and peaks at 2θ = 19.5°, 37.7°, 45.8°, and 67.0° corresponding to the gamma phase of Al_2_O_3_. The peak at 2θ = 30.2°, which is associated with the tetragonal phase of ZrO_2_, is particularly noted for its role in providing active sites for the esterification of oleic acid. The average crystallite size of the ZrO_2_/Al_2_O_3_ catalyst was determined using the Scherrer Equation which is given by:

𝐷=𝐾𝜆\𝛽cos𝜃.

Where:

D is the average crystallite size.

K is the shape factor (usually taken as 0.9).λ is the X-ray wavelength (for Cu Kα radiation, 𝜆=1.5406 A˚).

β is the full width at half maximum (FWHM) of the peak (in radians).

𝜃 is the Bragg angle (in radians).

For ZrO_2_/Al_2_O_3_ catalyst, the peak at 2θ = 30.2° for the calculation. The FWHM (β) for this peak obtained from the XRD data, is 0.5° (0.00873 radians).

θ = 2\30.2°=15.1°.

Convert θ to radians:

θ = 15.1°× 𝜋\180 = 0.263 radians.

By using the Scherrer Equation:

D = 0.9 × 1.5406\0.00873×cos (0.263) ≈ 165.88 A˚ ≈16.6 nm.

Thus, the average crystallite size of the ZrO_2_/Al_2_O_3_ catalyst, calculated from the Scherrer Equation, is approximately 16.6 nm, indicating a well-defined crystalline structure.

**Fig. 2:**
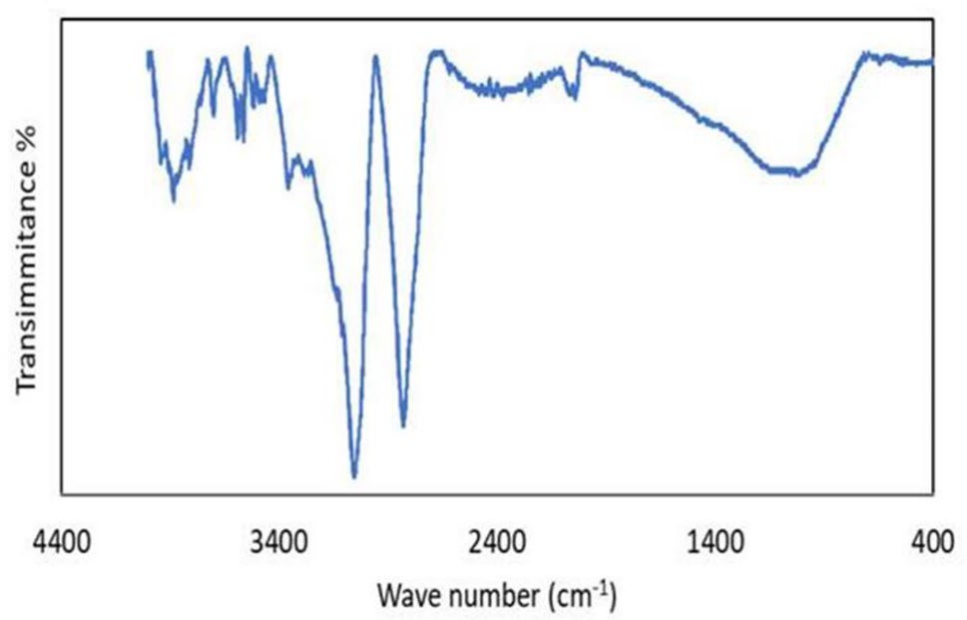
The ZrO_2_/Al_2_O_3_ IR spectra

The ZrO_2_/Al_2_O_3_ IR spectra are displayed in Fig. 2. Peaks in the range of 400–800 cm⁻¹ are often attributed to Zr-O and Al-O stretching vibrations. Bands around 1650 cm⁻¹ and 3400 cm⁻¹ can be assigned to bending and stretching vibrations of hydroxyl groups (OH) on the surface of the catalyst. These groups can play a role in the adsorption of reactants and the overall catalytic activity. Peaks around 1400–1500 cm⁻¹ might indicate carbonate species (CO_3_²⁻) adsorbed on the catalyst surface [27–29].

**Fig. 3:**
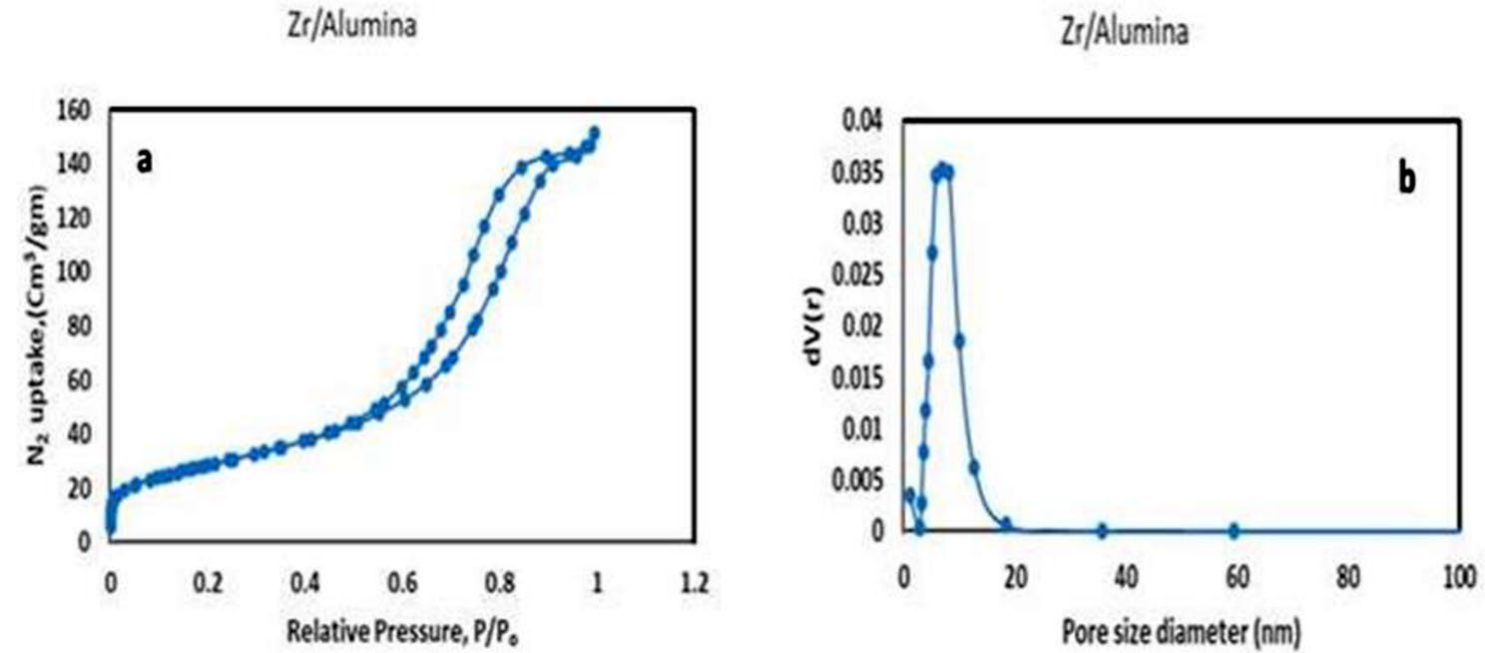
**a** & **b** The BET isotherm for a ZrO_2_/Al_2_O_3_

The BET isotherm for a ZrO_2_/Al_2_O_3_ catalyst follows a Type IV classification, exhibiting a characteristic hysteresis loop at higher relative pressures displayed in Fig. 3a & b. At low relative pressures (P/P₀ < 0.1), the isotherm shows a gradual increase in adsorption due to monolayer-type adsorption on the catalyst surface. As the pressure increases, the isotherm exhibits a steeper rise, indicating multilayer adsorption on the outer surface and pore walls of the ZrO_2_/Al_2_O_3_. At higher relative pressures (P/P₀ > 0.4), the hysteresis loop appears. This loop is attributed to capillary condensation within the mesopores of the catalyst. The adsorption branch of the loop represents the filling of pores with nitrogen, while the desorption branch shows the emptying of pores as the pressure decreases. Furthermore, the H3 hysteresis loop suggests that the ZrO_2_/Al_2_O_3_ particles aggregate into plate-like structures, resulting in the formation of slit-shaped pores. The observed pore size distribution with multiple peaks indicates potential irregularities in the arrangement of these slit-shaped pores, possibly due to the aggregation process [30–32].

**Fig. 4:**
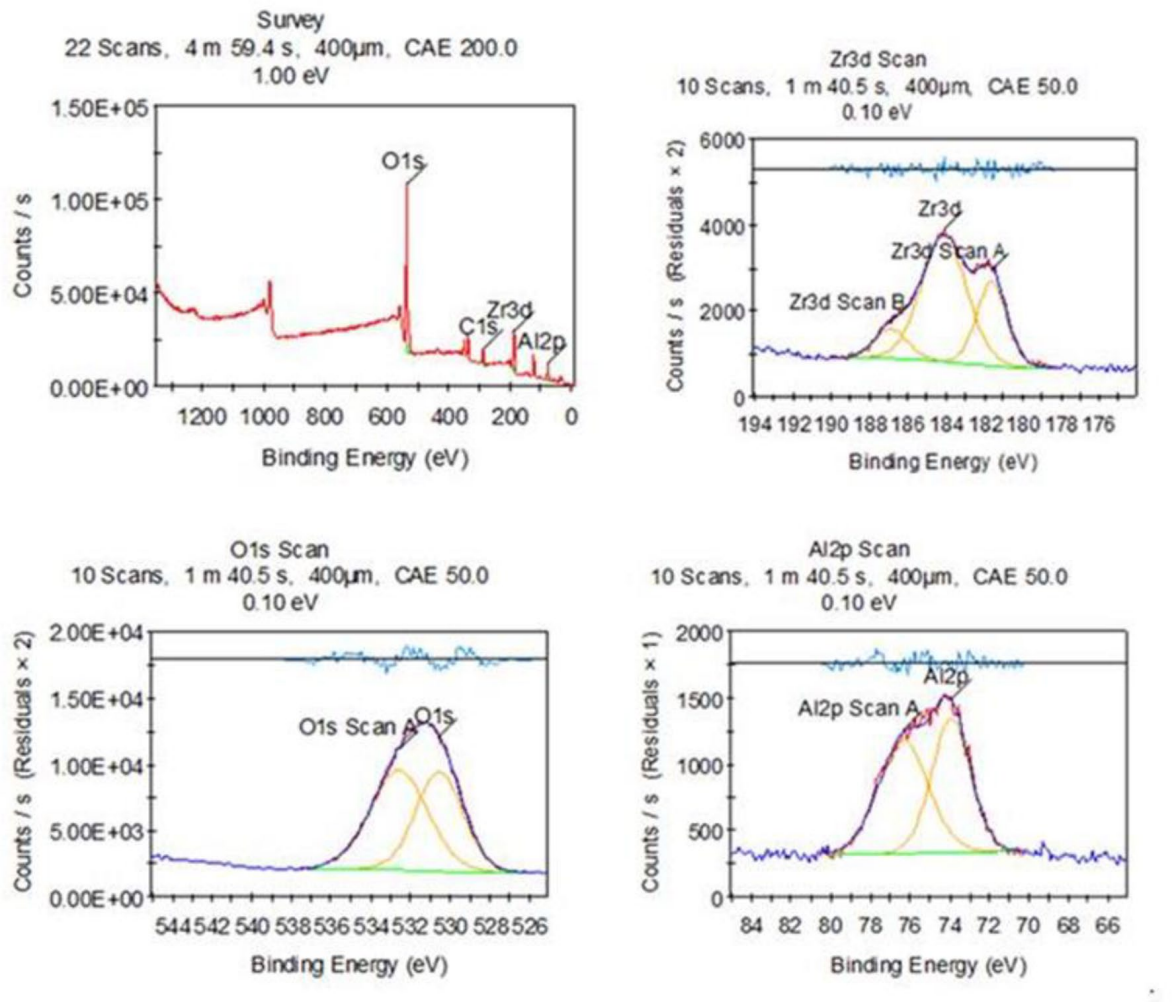
XPS of ZrO_2_/Al_2_O_3_ catalysts

X-ray photoelectron spectroscopy (XPS) is a powerful tool for analyzing the surface chemistry of ZrO_2_/Al_2_O_3_ catalysts Fig. 4. By examining the binding energies of core electrons, XPS provides valuable insights into the oxidation states and surface composition. The Zr 3d spectrum shows a main peak at approximately 182.2 eV, corresponding to the Zr 3d⁵/₂ electrons in Zr⁴⁺ of ZrO_2_. A smaller peak at a lower B_E_, around 180.5 eV, is likely due to Zr 3d^3^/_2_ electrons, also indicative of Zr⁴⁺. The absence of a significant peak at even lower B_E_ around 178 eV suggests a minimal presence of Zr⁰, which could be associated with oxygen vacancies. The Al 2p spectrum exhibits a peak centered around 74.5 eV, consistent with Al 2p electrons in Al³⁺ of Al_2_O_3_. Moreover, the broad O 1s peak centered around 532.8 eV can be deconvoluted into multiple peaks representing different oxygen environments. The peak at around 532.8 eV likely corresponds to lattice oxygen in ZrO_2_ and Al_2_O. A shoulder at a higher binding energy at about 534 eV could be attributed to surface hydroxyl groups (OH^−^) [33, 34].

**Fig. 5:**
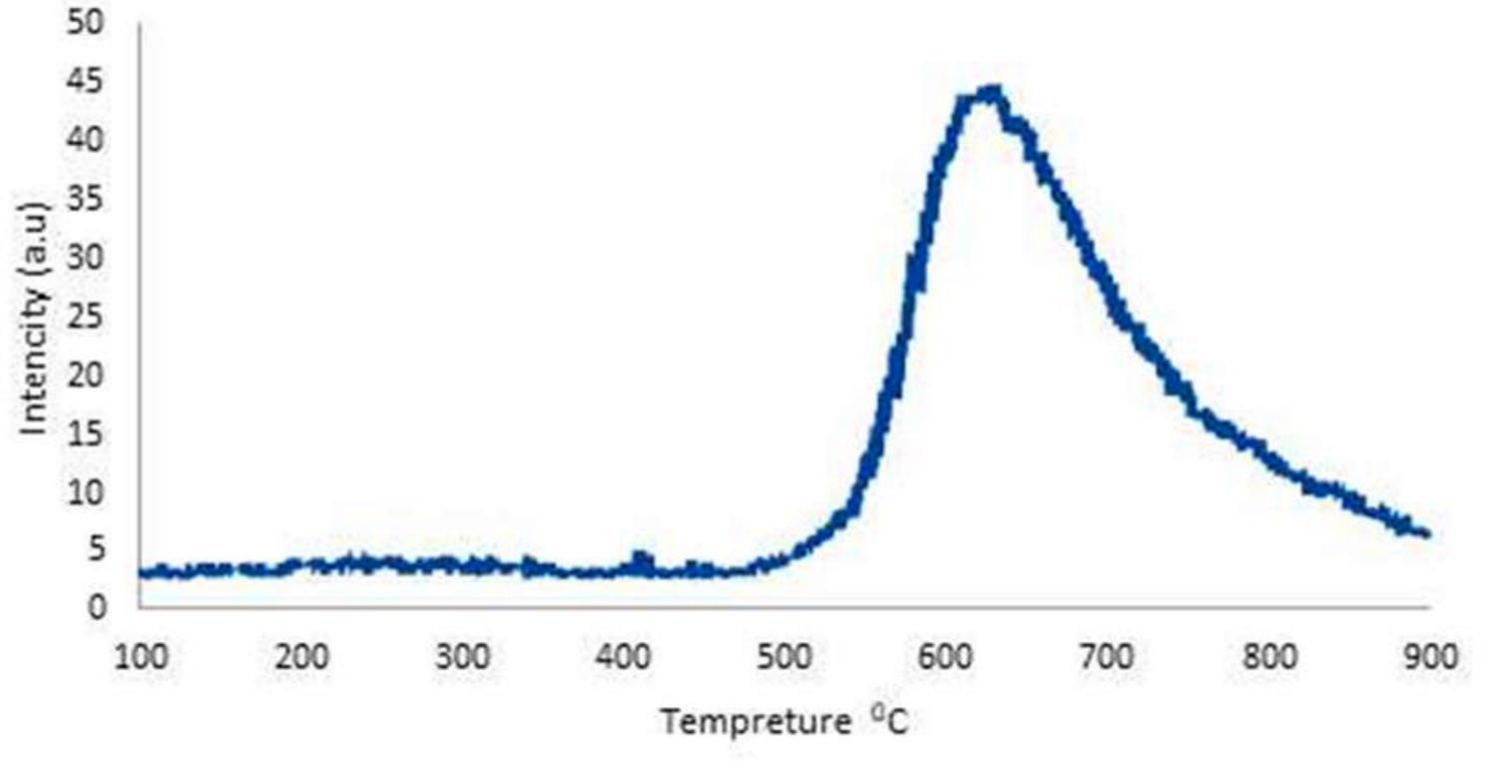
Temperature programmed desorption TPD-NH_3_ of ZrO_2_/Al_2_O_3_

The observed TPD-NH_3_ Fig. 5 exhibits a broad peak with a maximum desorption rate of around 620 °C with a total acidity of 0.635 mmol/g indicating a distribution of acid sites on the ZrO_2_/Al_2_O_3_ surface with a significant portion exhibiting strong interactions with NH_3_ molecules [35, 36]. 

### Catalytic formation of ethyl oleate

**Fig. 6:**
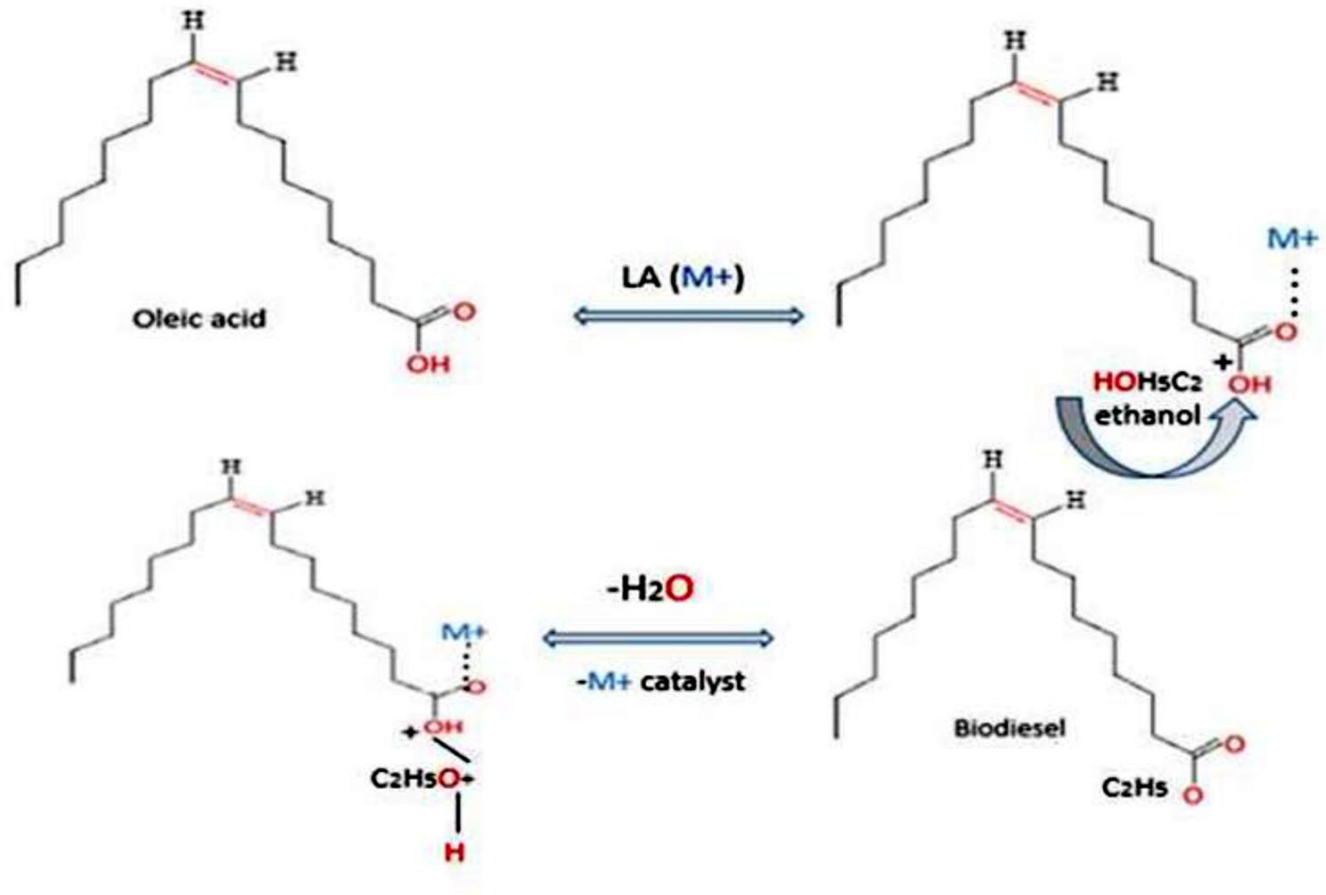
Catalytic formation of ethyl oleate by Lewis acid ZrO_2_/Al_2_O_3_

The catalytic formation of ethyl oleate was described in Fig. 6. As shown, the initial step of the catalytic esterification involves the Lewis acid sites on ZrO_2_/Al_2_O_3_ interacting with the carbonyl oxygen (C = O) of oleic acid. This interaction weakens the O-H bond in the acid, facilitating its protonation by ethanol and the formation of a carbenium ion intermediate. Subsequently, the alcohol moiety from ethanol adds to the carbenium ion, and a water molecule departs, leading to a structural rearrangement that yields the final ester molecule.

### Acid value reduction

The acid value of the oleic acid sample was measured to evaluate the effectiveness of the esterification process catalyzed by ZrO_2_/Al_2_O_3_. The initial acid value of the oleic acid was 200 mg KOH/g, indicating a high free fatty acid content. After the esterification reaction, the acid value significantly decreased to 5 mg KOH/g, demonstrating a high conversion efficiency. This substantial reduction in acid value confirms the catalyst’s capability to convert free fatty acids into esters effectively, thereby enhancing the quality of the produced biodiesel.

### FTIR analyses

**Fig. 7:**
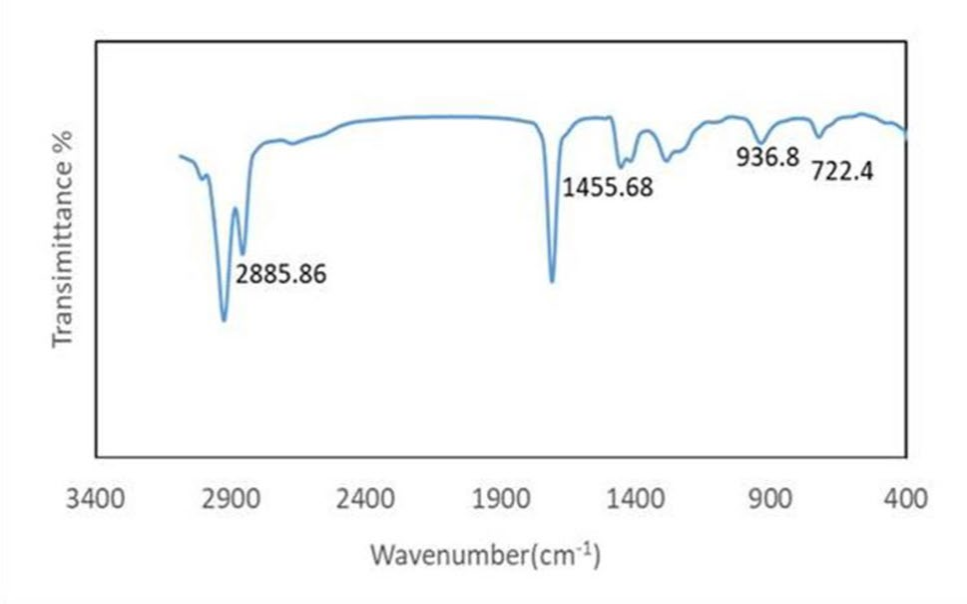
FTIR of oleic acid

**Fig. 8:**
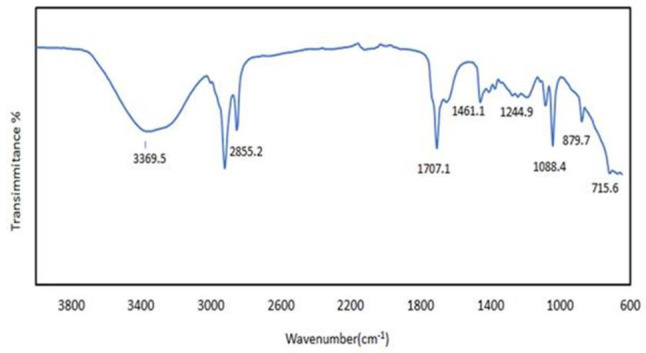
FTIR of biodiesel

To confirm the conversion of oleic acid to biodiesel through the esterification process, FT-IR analysis was performed on both samples. Figures 7 and 8 illustrate the resulting spectra obtained in the broad wavelength region of 400–4000 cm⁻¹. The spectrum of oleic acid exhibits characteristic peaks for the following functional groups (Fig. 7) where strong, broad peaks due to stretching vibrations of C-H bonds in the long aliphatic chain of oleic acid at (3000 –2800 cm⁻¹). (1710 –1700 cm⁻¹) C = O stretch carboxylic acid sharp, intense peak indicating the presence of the carbonyl group (C = O) in the carboxylic acid moiety of oleic acid. Around (1450 cm⁻¹) C-H bend (alkenes) bending vibration of C-H bonds adjacent to the double bond (C = C) in the cis configuration of oleic acid. The FT-IR spectrum of the ester (Fig. 8) shows the disappearance of the carboxylic acid peak and the emergence of new peaks characteristic of the ethyl group, along with the presence of ester functional group vibrations. This confirms the successful conversion of oleic acid to ethyl oleate through esterification with ethanol. Strong, broad peaks due to stretching vibrations of a long aliphatic chain of oleic acid C-H bonds around (3000 –2800 cm⁻¹). Asymmetric stretching of CH₂ groups in the ethyl moiety (C₂H₅) on about (2980 cm⁻¹). Symmetric stretching of CH₂ groups in the ethyl moiety at (2930 cm⁻¹). Symmetric stretching of the CH₃ group in the ethyl moiety at (2870 cm⁻¹). C = O Stretch sharp, intense Ester peak (1740 –1730 cm⁻¹) in this region signifies the presence of the carbonyl group (C = O) in the newly formed ester linkage. This peak is similar to the one observed in oleic acid but might show a slight shift in position [37, 38].

### Influence of esterification reaction parameters on the catalytic performance of ZrO_2_/Al_2_O_3_

#### Effect of molar ratio of ethanol to oleic acid

**Fig. 9:**
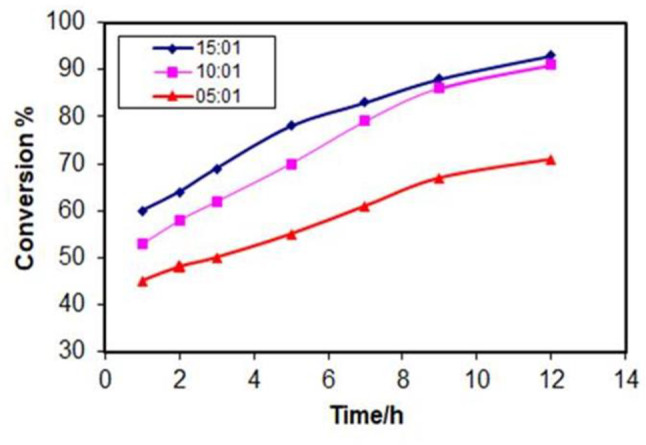
Effect of molar ratio of ethanol to oleic acid

The molar ratio of ethanol to oleic acid is a critical parameter affecting ester yield. Here, all other reaction conditions were held constant while the molar ratio was varied from 5:1 to 15:1. As displayed in Fig. 9, conversion of oleic acid increases with increasing ethanol to oleic acid ratio of about 95% this is due to a higher concentration of ethanol increases the probability of collisions between ethanol molecules and oleic acid molecules, leading to a faster reaction rate and higher conversion of oleic acid. Also from Fig. 9, we noticed that the conversion of oleic acid reaches a plateau at a molar ratio of around 10:1(95%). This suggests that there is an optimal ratio of ethanol to oleic acid for this reaction. Beyond this point, increasing the ethanol concentration has little to no effect on the conversion (about 1%). This could be due to there being a limited number of active sites on the ZrO_2_/Al_2_O_3_ catalyst that can bind to oleic acid molecules. Once all the active sites are occupied, adding more ethanol won’t increase the reaction rate and the esterification reaction is reversible, meaning that some of the ethyl ester can react with water to form oleic acid and ethanol again. At higher ethanol concentrations, the reverse reaction may become more favorable, leading to a decrease in the overall conversion of oleic acid [39].

#### Effect of catalyst dosage

**Fig. 10:**
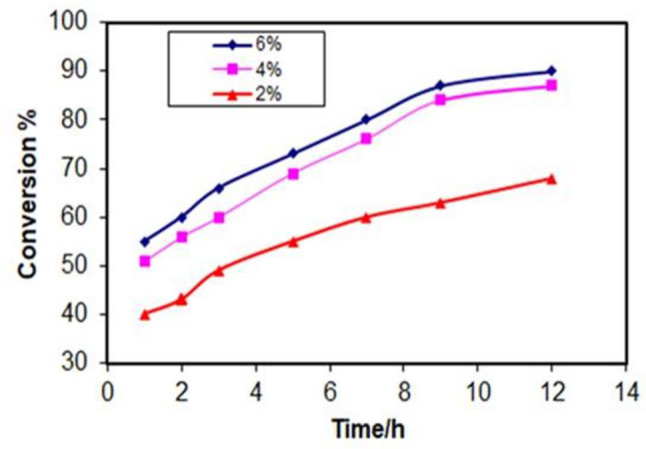
Effect of catalyst dosage

From Fig. 10, as we added more catalysts, there were more active sites available for the reaction to occur, leading to a faster conversion rate and higher yield of the product (ethyl oleate) at a given time. Also, we noticed that the increase in conversion between 4% and 6% catalyst loading is smaller compared to the increase between 2% and 4%. This could be due to higher catalyst concentrations, all or most of the catalyst sites might be occupied by reactant molecules. Adding more catalysts won’t significantly increase the number of collisions between reactant molecules, limiting the further increase in conversion rate and also it could be due to the reactant molecules having difficulty reaching the available catalyst sites due to the increased viscosity or limitations in mass transfer within the reaction mixture. This means that 4% is optimal for this process [40, 41].

#### Effect of reaction temperature

**Fig. 11:**
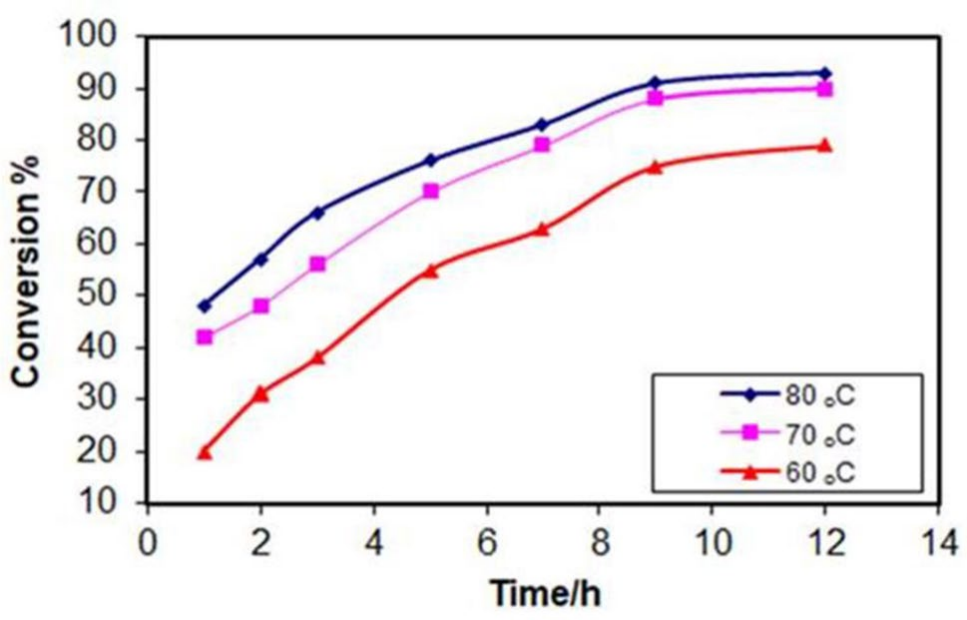
Effect of reaction temperature

Figure 11 shows the effect of reaction temperature (60 °C, 70 °C, and 80 °C) on the conversion rate of esterification reaction of oleic acid to ethyl oleate. The conversion rate increases with increasing reaction temperature. This is a common observation in many chemical reactions, and it follows the principles of reaction kinetics [42]. Higher temperature increases the average kinetic energy of the reactant molecules, making them move faster and collide more frequently. This leads to a greater chance of successful collisions between oleic acid and ethanol molecules, resulting in a faster conversion rate of oleic acid to ethyl oleate [43]. Esterification is an equilibrium reaction, meaning it can proceed in both the forward and reverse directions. At higher temperatures, the equilibrium might shift towards the reverse reaction, reducing the final yield of the product (ethyl oleate). High temperatures can sometimes favor unwanted side reactions, leading to the formation of undesired products and reducing the overall efficiency of the process. Considering economic factors, a reaction temperature of 70 °C appears most favorable based on the achieved conversion rate [44]. This temperature will be used for further parameter verification, as the conversion efficiency at 70 °C approaches that of 80 °C while potentially offering a higher final yield of ethyl oleate due to reduced side reactions at the lower temperature.

### The reusability of the catalyst

**Fig. 12:**
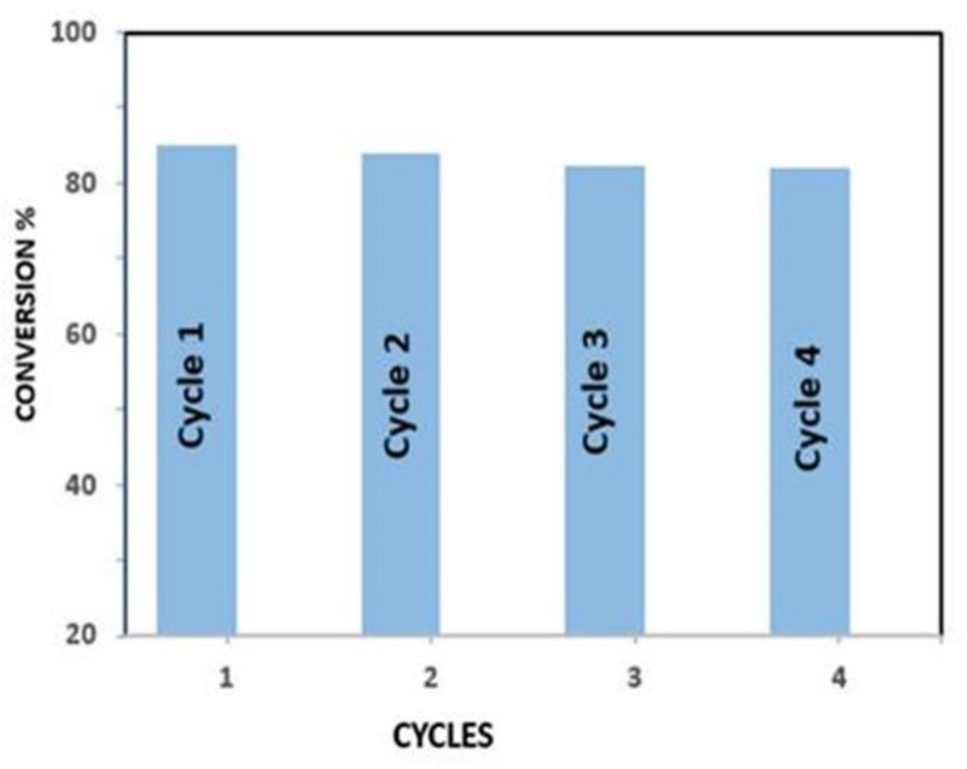
The reusability of the catalyst

The ZrO_2_/Al_2_O_3_ catalyst exhibits promising reusability for the esterification reaction (Fig. 12). The conversion rate of oleic acid remains acceptable after four cycles, indicating that the catalyst can be reused multiple times without significant activity loss. Furthermore, the catalyst demonstrates the potential for recyclability. A simple regeneration process after each cycle allows the catalyst to maintain its conversion efficiency up to the fourth cycle. However, a slight decrease of around 3% in conversion is observed by the fourth cycle.

### Comparison with other solid acid catalysts

**Table 1:** Comparison of ZrO_2_/Al_2_O_3_ with other solid acid catalysts

Catalysts	Temperature (C^o^)	A/O Molar ratio	Catalysts loading (wt %)	Oil Conversion (%)	Reusability	Ref.
ZrO_2_/Al_2_O_3_	70	10:1	4	90.5	4 cycles	This work
SO_4_^− 2^ /ZnO	65	6:1	4	80.2		[45]
ZrO_2_ –Supp. AC zeolite	65	10:1	10	84.2		[46]
SO_4_^− 2^ / ZrO_2_- Al_2_O_3_	180	8:1	6	83.8		[47]
AC from Corncob	75	6:1	20	88.7		[48]

The comparison of catalytic performance and reusability of the synthesized ZrO_2_/Al_2_O_3_ catalyst with other solid acidic catalysts reported in the literature for biodiesel production is challenging due to the different reaction conditions employed. Nevertheless, as indicated in Table 1, the ZrO_2_/Al_2_O_3_ catalyst demonstrated superior or at least comparable catalytic activity and stability. This indicates that the well-designed structure and synergistic effects between ZrO_2_ and Al_2_O_3_ effectively enhance the transesterification performance of the fabricated solid catalyst. Therefore, the ZrO2/Al2O3 catalyst is an efficient and durable option for cost-effective biodiesel production, especially when using acidic oils as feedstocks.

## Conclusion

This study successfully synthesized and characterized a novel ZrO_2_/Al_2_O_3_ heterogeneous catalyst using the sol-gel technique and investigated its applicability for the esterification of oleic acid to ethyl oleate (biodiesel). The catalyst was characterized using various techniques, and its performance was evaluated under different reaction conditions. The XRD analysis revealed characteristic peaks of the tetragonal phase of ZrO_2_ and the gamma phase of Al_2_O_3_, with the average crystallite size calculated to be approximately 16.6 nm. BET surface area analysis demonstrated the catalyst’s high surface area and mesoporous structure, which are crucial for catalytic activity. FTIR and XPS analyses confirmed the presence of essential functional groups and the chemical states of the elements, respectively. NH_3_-TPD analysis indicated a broad distribution of acid sites, essential for the esterification reaction.

Under optimized conditions (70 °C, 10:1 alcohol-to-oleic acid molar ratio, and 4 wt% catalyst loading), the catalyst achieved a remarkable 90.5% conversion of oleic acid to biodiesel. Furthermore, the catalyst exhibited significant reusability, maintaining its activity over multiple cycles, thus demonstrating its potential for sustainable biodiesel production from low-grade oleic acid feedstock. The comprehensive characterization of the ZrO_2_/Al_2_O_3_ catalyst and its high catalytic performance underscore its applicability in biodiesel production, providing a viable route for renewable energy generation. Future research should focus on optimizing the catalyst synthesis process to further enhance its activity and stability. Exploring the use of other abundant and low-cost feedstock for biodiesel production could also broaden the applicability of this technology. Additionally, investigating the reaction mechanisms in greater detail through advanced characterization techniques would provide deeper insights into catalyst behavior and performance.

Moreover, integrating biodiesel production with other renewable energy processes, such as bioethanol production from agricultural waste, could create a more sustainable and circular economy. Pilot-scale studies and life cycle assessments are essential to evaluate the environmental and economic feasibility of these technologies on an industrial scale.

## Data Availability

No datasets were generated or analysed during the current study.
